# Twitter as a Potential Disaster Risk Reduction Tool. Part I: Introduction, Terminology, Research and Operational Applications

**DOI:** 10.1371/currents.dis.a7657429d6f25f02bb5253e551015f0f

**Published:** 2015-06-29

**Authors:** Guy Paul Cooper, Violet Yeager, Frederick M. Burkle, Italo Subbarao

**Affiliations:** College of Osteopathic Medicine, William Carey University, Hattiesburg, Mississippi, USA; College of Osteopathic Medicine, William Carey University, Hattiesburg, Mississippi, USA; Harvard Humanitarian Initiative, Harvard University, Cambridge, Massachusetts; The Woodrow Wilson International Center for Scholars, Washington, DC, USA; College of Osteopathic Medicine, William Carey University, Hattiesburg, Mississippi, USA

**Keywords:** Communications, Disaster risk reduction, Prevention and preparedness, Social media, Twitter

## Abstract

Twitter, a popular communications platform, is identified as contributing to improved mortality and morbidity outcomes resulting from the 2013 Hattiesburg, Mississippi EF-4 Tornado. This study describes the methodology by which Twitter was investigated as a potential disaster risk reduction and management tool at the community level and the process by which the at-risk population was identified from the broader Twitter user population. By understanding how various factors contribute to the superspreading of messages, one can better optimize Twitter as an essential communications and risk reduction tool. This study introduces Parts II, III and IV which further define the technological and scientific knowledge base necessary for developing future competency base curriculum and content for Twitter assisted disaster management education and training at the community level.

## INTRODUCTION

With the advent of new technologies, our ability to communicate with one another has evolved significantly. No longer are societies solely dependent on traditional media outlets, newspapers, radio, and TV for the news. With rapidly evolving smartphone technologies, societies are just an ‘app’ away from being able to deliver or receive information within milliseconds. Popular social media platforms such as Twitter, Facebook, and YouTube, have supplanted the traditional media outlets for accessing and responding to information. Every day millions of users worldwide are connected and receive their news via online social networks warranting researchers to study the mechanisms behind human interactions.^1^

Recently, Twitter has been used for spreading news and updates around the world and has been shown to have application in emergency situations^2^ of natural disasters such as earthquakes^3^^–^ 5, floods^6^^–^ 8, hurricanes^9^^,^ 10, and wildfires.^11^ Twitter has shown to have the potential to increase survival during Tornado-related disasters.^12^ Social media’s technology platforms allow for multidirectional network communication which can aid officials during disasters to compile a list of the injured, deceased, and contact family and friends of victims^13 ^ all while connecting and organizing both casualties and responders.^14^^–^ 26 This provides public and mental health value to the population affected by connecting vital services and resources.^27^^–^ 29

What has not been explored adequately is whether these new platforms can be recognized as vital resources to improve response to disaster and crisis events by targeting a specific geographic location that is susceptible to a disaster. The inability to reach geographically targeted populations remain some of the main reason for inadequacies in disaster response, especially where critical information needs to reach and be disseminated rapidly to the most ‘at-risk and vulnerable populations’.^30^^,^ 31 Despite communication channel disruption during Japan’s 2011 Tohoku tsunami and earthquake, Twitter facilitated real-time communication with emergency management officials and new agencies.^14^^,^ 32

Disaster managers constantly work to discover ways to mitigate morbidity and mortality throughout the entire disaster cycle: prevention, preparedness, response and recovery. This capacity is largely dependent on community empowerment and community level mobilization and health promotion efforts.^33^ Verma wisely reminds us that despite multiple international initiatives over that past several decades directed toward disaster risk reduction, communities of “people who are at the bottom of the pyramid of preparedness for disasters” must be empowered and given the opportunity to be “made partners in the process of preparedness with usable knowledge, social awakening, practical training and guidance for use of local resources indicatively to respond with developed skills.”^34^ Social media has the potential for information interconnectivity, reliability, and increasing breath for information all of which are important features for engaging the target population.

Twitter has become an immensely valuable tool worldwide, particularly in disseminating and conversing about issues in everyday life. Despite a limited 140 character maximum and the consequential pithy form of text used, it remains an extremely popular means of communication. It is estimated that there are over 517 million Twitter users worldwide with approximately 142 million that reside in the U.S. alone.^35^

There has been a growing list of peer-reviewed publications on the use of Twitter for emergency situations, however there is a glaring lack of understanding of the technological base and science behind Twitter usage at the operational and practical application level. This study has a two-fold goal, one to introduce Twitter as a viable communications tool at the community level during crisis events with potential for disaster risk reduction and management, and two to establish an evidence-based technological science and knowledge base necessary for community-level replication and education and training of this communications tool.

**BASICS OF TWITTER USE**

Twitter, as have other commonly used programs, has acquired its jargon or specialized language using slang terms that may not be readily understood by the uninitiated reader (Figure 1, Table 1). On any one day over 500 million tweets, or messages, are made.^36^ To become a Twitter user, one needs to register creating a Twitter profile. The profile is publically accessible and contains a section of biography and location, although there is no requirement for a Twitter user to upload accurate information into their profile.

An average Twitter user or sender has 208 followers; once a tweet is sent it becomes immediately visible to all followers (Figure 2).^37^ If the message is found worthy enough, it can be retweeted on to secondary followers. This occurs either as a pure/exact retweet, meaning the entire tweet is re-messaged forward in a precise manner by one of the secondary followers, or as a “modified retweet,” where a part of the tweet is forwarded or what is known as a “user mention” tweet where only the original Twitter username or “handle” is transmitted (Figure 2, Table 1). The original Twitter “handle” may be mentioned in any subsequent tweets, so it is easily traceable back to the original message (Figure 2).

**Screen capture of a Twitter user home page with labels.**
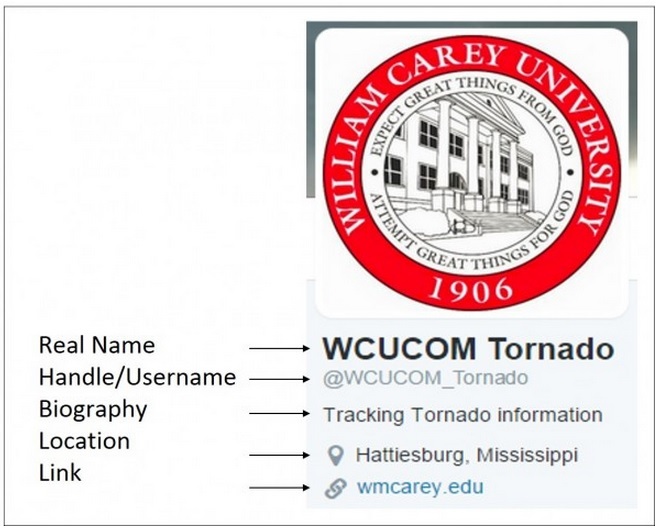

**Table 1. Commonly used Twitter terms.** 

Term	Definition
Klout	A commercially manufactured metric that ranges from 1-100 which measures and rates the 'social influence' of an individual user on Twitter.
Tweet	140 character publicly viewable message that may additionally contain links and images.
Trend	A topic that is currently being heavily discussed on Twitter.
User mention (UM)	Another Twitter user is mentioned by name in your tweet.
Retweet (RT)	A specific tweet that is duplicated by another user.
Follower	A user that is notified or updated every time the followed user posts a tweet.
Bot	A Twitter account operated by an automated software application.
Twitter: Username (@)	Also called a Twitter handle, a unique Twitter identity.
Twitter: Real Name	The name a user labels himself on Twitter (not necessarily unique).
Twitterverse	The defined Internet space where all tweets and users exist on Twitter.
Hashtag (#)	A symbol used to mark keywords or topics in a tweet. It allows a tweet to be seen globally on Twitter.
Twitter Profile: Location	A free text area for a Twitter user to input his location, but it is neither verified nor required.
Twitter Profile: Biography	A free text area for a user to input his biography, but it is neither verified nor required.
Meme	A transmissible unit of information phrase or links such as a hashtag.^38^

**Sample screen capture of a Tweet, User Mention, and Retweet.**
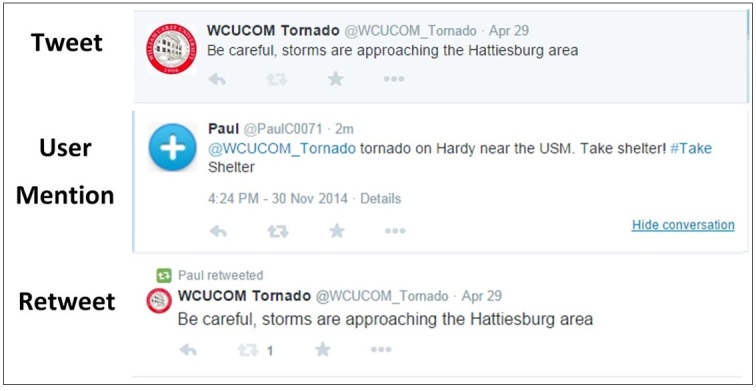

Once a message is retweeted either in an exact manner or user mentioned it becomes visible to their multiple new followers. This visibility allows for an exponential sharing of information that in turn can lead to a ‘viral’ dissemination of the original message. This viral propagation results in messages being widely popular, referred to in the Twitter jargon as a “trend” spread, becoming visible even by non-followers and by those at large on the Internet. Previous studies have shown that 16% of tweets are retweeted.^39^ However, this viral communication is not just one way, in fact, the inherent process allows for a robust bi-directional method of communication between Twitter users. Also, any retweet demonstrates to other users a cognitive verification that the message was received and found to be of personal value. Twitter purposely uses ‘Hashtags’ as part of the tweet as it serves as one of the most powerful means of enhancing exponential messaging. Hashtags permit anyone on the Internet (followers and non-followers) to access the tweet and to disseminate messages across newly defined electronic communities without restrictions or borders. Naturally, the use of hashtags enhances the opportunity for a tweet to go viral. Twitter also allows for the storing of tweets and will further publicly stream every tweet and its associated metadata of information. This metadata includes 20 fields of data that are sorted into three primary categories: location (including the time zone), biography (occupation-personal interest), and the tweet messages themselves. Additionally, Klout scores are a commercially manufactured metric that measures and rates the 'social influence' of an individual user on Twitter. (Table 1) The Klout metric score takes into account many factors including the number of times the user’s message is retweeted and the number of times the tweet is reintroduced or mentioned (user-mentions).^40^ Simply starting a Twitter account and tweeting does not guarantee that the message will reach its intended audience or in the case of a crisis the ‘at-risk or vulnerable population’.

It is known that less than 2% of individuals enable the GPS feature during the course of regular use.^41^ Turning it on only at the time of a disaster may be too late. Furthermore, Twitter account users are not required to provide accurate profile information to start up an account. Users may do this intentionally for anonymity and privacy.

**TWITTER AS A POTENTIAL DISASTER COMMUNICATIONS TOOL**

Unfortunately, these properties of Twitter make it difficult to know whether a purposeful tweet was received and followed by the targeted audience and remains a major factor limiting its potential use as a disaster communications tool. Therefore, the question remains on how to identify the at-risk population from within the hundreds of millions of Twitter accounts that currently exist. In this study the authors refer to finding the one elusive “haystack” among millions of Twitter handles as the at-risk population that would benefit from this communication at the time of a disaster. If identified, the question remains on how can the process assure they are targeted accurately in the future? If the at-risk population or haystack can be defined, could this process also serve to monitor communities through both active and passive surveillance crucial in crisis analysis to show outcome performance? In this process, the at-risk population (the population numerator) and the total geographic population of all users (the population denominator) are necessary. Only then can one begin to identify the characteristics and qualities of individuals that are retweeted by the haystack. This process will then allow disaster managers to better identify the most modifiable variables that could influence the required “superspreading” of the vital disaster messages to the at-risk populations which, in this analogy, becomes the ‘needle in the haystack.’

By identifying these essential qualities and characteristics of the population, one can purposefully employ Twitter in a targeted and directed manner during any crisis. The apparent benefit of such a process is to rapidly warn and direct the population to immediately seek safety and to assist disaster managers in resourcing those areas and individuals most vulnerable and in need. A critical goal of this study is to provide a means for other investigators and disaster managers to utilize Twitter as a prevention tool for their communities. The authors offer two additional studies (Parts II and III) to describe the technological process by which one can identify both the ‘haystack and the needle populations at-risk’. In doing so, the study utilized as a working template the 2013 Hattiesburg, Mississippi tornado, a large and violent EF4 multiple vortex tornado that devastated wide portions of West Hattiesburg and surrounding communities.^12^

**TWITTER DATA ANALYSIS: PREVIOUS METHODOLOGICAL APPROACHES**

A recent exhaustive review of existing methodological approaches for Twitter data analysis including public health and disaster practice demonstrated inadequate methods for sampling, particularly for regional locations.^42^ The most common method for Twitter data analysis focuses retrospectively on a single or group of keywords, hashtags, or users and does not take into account geographical location, but rather general Twitter trending. Ruths and Pfeffer argue for the need for better methodological approaches, highlighting the common errors of data interpretation specifically due to inaccurate sampling and other inference errors, particularly those due to reliance exclusively on keywords topical searches alone.^42^ These studies do not accurately sample the regional population because they target the keyword, the numerator, without addressing the location, the denominator, which allows for the keywords to be driven by global rather than local changes. This approach can grossly overestimate the interpretation of keyword surveillance, for example ‘big error’ often plagues big data as was seen in the 2009 swine influenza pandemic.^43^^,^ 44

The challenge with GPS is that while it is excellent at identifying targeted geolocation tweets, most people prefer anonymity and do not turn the feature on. An advanced approach often utilizes real-time filtering of the Twitter ‘firehose’ through Twitter’s publicly available interface, but this only represents 1% of current activity on Twitter.^45^ This interface (Twitter API, application programming interface) has strict limitations on the quantity of tweets that can be collected in a given timeframe. The advanced approach ends up dealing with big data which is difficult to store and represents only a small sample size due to Twitter’s limits for acquiring tweets. Third party sources can be utilized to access large volumes of data, but can be costly and require programming skills to access and analyze these large sums of data. The main limitation of all the Twitter studies is in the validity and reliability of these approaches to accurately sample and identify geographically at-risk ‘regional users.’ Weng and colleagues are advancing the predictability of successful memes using their early spreading in the underlying social networks and by analyzing a “comprehensive set of features to develop a model to predict future popularity of a meme given its early spreading patterns.” 46

## Proposed Methodology to Identify Regional Users

A novel two-step method is proposed to overcome these challenges. The first step includes data extraction of all tweets utilizing PowerTrack rules from GNIP (an authorized reseller of Twitter data) that include broad-based tornado disaster centric filters.^47^ The second step uses a triangulated approach to filter and identify regional Twitter users. The methodology utilizes a filtration approach to ‘capture or triangulate’ tweets from multiple angles (location, biography, retweets of Twitter users) to ensure they were actually utilized by the at-risk disaster affected geographic population (Figure 3).

**Regional Triangulation Model:**
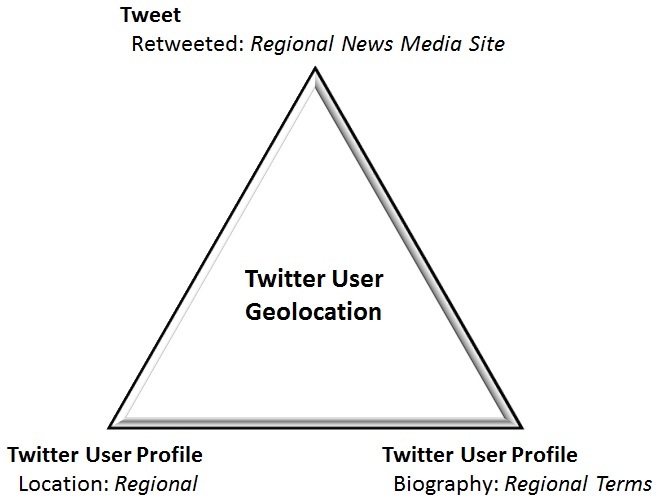

**TRIANGULATION OF REGIONAL TWITTER USERS: IDENTIFYING THE DENOMINATOR**

Triangulating essentially means using three publically accessible variables, the tweet itself, the Twitter profile location metadata, and the Twitter profile biography metadata as a proxy for geolocation—to best identify that a Twitter user is from the geographically at risk region (Figure 3). The likelihood of a retweet of a regional news media outlet (TV, Radio, Newspaper) would be a strong proxy for someone with ties to the area. The Pew Research Center study recently showed that 50% of Twitter users use the tool primarily for news.^1^ Having named a geographically unique term to the region including universities in either their Twitter Profile Biography or Location also would increase their likelihood to the area. Regional filters were then applied to the extracted data based on the users: tweets, biographies, and locations (Figure 2). The categories were then evaluated to determine the contribution of each of the filters.

**VALIDATION OF METHODOLOGY**

A team of researchers with public health epidemiology background, independent from the coding team, evaluated the data to determine if regionality was included in the criteria established in Table 1 and if non-regionality was appropriately excluded. Regional users were confirmed and validated in a two-tier approach based upon the available GPS coordinates and an independent quality assessment of individual Twitter users and tweets. GPS coordinates for users that had activated their geo-locations were compared against regionally defined users to confirm their presence in Alabama or Mississippi. GPS sample standards were set as a 99% confidence interval and a 3.0% margin of error (sample size of 900 users).

The study received an IRB exemption for human subject research from the William Carey University IRB Committee.

## Results

We developed a novel methodological framework for identifying geographically located twitter users using geographically unique regionally specific metadata parameters. This approach essentially allows us to identify twitter users in the region that do not have their GPS turned on. Table 2 summarizes the research questions and the methodology used to address these essential issues.

**Table 2. Summary of the research questions and the methodology.** 

Part	Research Question	Methodological Approach
I	Sampling bias often plagues regional tweet and Twitter user analysis.	Describes the development of a new methodological approach for identifying location sampling of geographically defined Twitter users.
II	Unique triangulation approach needs sample application and validation to be shown useful for practical application.	Practically applies and validates the user Triangulation method to the 2013 Hattiesburg EF-4 Tornado Twitter users.
III	Regional data is useful but requires further analysis to exhibit meaning to variables.	Analysis was performed on modifiable and non-modifiable variables associated with the locally influential users.
IV	Regional variables can be established, but for practical implementation to occur established standards must be met.	Proposes social media disaster communication core competency changes in current public health disaster management education for a practical application while staying true to currently established standards.

Parts II and III further define the specific technical aspects of the proposed methodology using the 2013 Hattiesburg Tornado template and serves as a follow-up to an earlier study that confirmed a decreased morbidity and mortality rate as compared to prior storms of similar tornado risk.^12^ Paper II describes the detailed methodological efforts used to discover the haystack of tweets transmitted during the 2013 Hattiesburg Tornado among those captured from the over 2 billion tweets in the 96 hour window of the storm that were emitted by the Twitter system.^48^ The data generated from the approach provides a descriptive analysis of the regional Twitter activity 48 hours pre- and post- Hattiesburg Tornado. Part III describes ‘the needle in the haystack’ by identifying the Top 100 Twitter Users that were retweeted 48 hours pre- and post- tornado and analyzing the significant statistical relationship between metadata variables and in particular hashtags of the users themselves.^49^ Additionally, the three-part study attempts to answer the question whether the regional community would find local organizations or individuals using Twitter as trusted disaster information communicators. The authors suggest that this will be realized through proper competency based curriculum content (Part IV) necessary for standards-based education and training at the community level.^50^

## Discussion

Traditional disaster communication technologies exist for tornado response but possess inherent limitations (Table 3). While all modern technologies require a device and internet, Twitter is uniquely designed for effective and efficient two way communication. Twitter is also cost effective, used daily by the community, and allows for broad dissemination. Twitter can also be used in all phases of tornado preparedness and response and is the simplest and most redundant communications tool. Twitter can, along with traditional communication approaches, ensure that every community is a prepared community.

**Table 3. Disaster Communication Tools:** 

Type	Technology	Limitations	Two-Way Communication	Mass Distribution	Pre-Tornado	Tornado	Post-Tornado
Traditional							
	911	Victims often overwhelm 911 dispatchers	X			X	
	Tornado Sirens	Not always available or audible		X		X	
	Local Radio	Requires radio and power (or batteries)		X	X	X	X
	Local Television	Requires television, cable (or antenna) and power (or batteries)		X	X	X	X
Modern							
	Local News & Weather Apps	Primary focus is on infrastructure damage and tornado pathway		X	X	X	X
	Disaster Phone Apps	Lack of familiarity due to infrequent use and primarily focuses on sheltering		X		X	X
	Facebook	Privacy restrictions may limit mass distribution and two-way communication	X	X	X	X	X
	Twitter	Users do not require validation	X	X	X	X	X

Currently, there are limited studies providing an easily accessible methodology to determine the effectiveness of Twitter for risk communications in a disaster.^13^ There has been an increased interest in utilizing Twitter and other social media technologies for disaster management.^51^ However, many studies focus on the situational awareness aspect for “numerator data information,” and inaccurately estimate the needs of the population affected. Twitter can contribute to a more accurate depiction of situational awareness via crowd sourcing and complement conventional approaches during a disaster or emergency by employing our methodological approach to gain more accurate population sampling. This approach triangulates geographic proxies from the metadata in the Twitter profile and circumvents the issue of GPS (less than 2% have it turned on) and has the potential to provide an accurate sample.^52^^,^ 53 This information gained via human sensor networks through Twitter can aid in establishing reliable situational awareness from the disaster affected area.^53^

A recent Congressional Service Report on the use of social media in disasters identifies best practices for risk communication during disasters consist of identifying the target audience, and in disseminating accurate and appropriate types of information.^13^ For effective Twitter use during a disaster, communication culture, competency, and credibility must be established.^32^^,^ 42 Location plays a fundamental role in meeting these criteria. The location also provides the ‘denominator data information’ from which accurate data assessment can be made about the actual needs of the population. For the application of Twitter in disaster communication, identifying and targeting the twitter users from the geographically susceptible area must be established to ensure they are active participants in the dialogue and response. While past studies have highlighted location through the use of “hard metadata” such as GPS^42^^,^ 43, we suggest the incorporation of regionally unique ‘soft metadata’ from the Twitter profile as a valid proxy source for location.

Only by providing a usable methodology for a local population or subset of a population can one best realize Twitter’s potential. Unfortunately, local communities have taken the short stick when it comes to effective disaster planning and prevention. Studies of influenza response over the last decade show that whereas tactical and strategic plans worked well there were significant operational “gaps, weaknesses, inconsistencies and failures” at the local community level.^54^ Furthermore, the United Nations Hyogo Declaration and Framework for Action of 2015 provides an evidence-based foundation in health that is considered essential in community-level communication. In doing so, the Framework highlights the need for governance of disaster risk reduction and resilience by establishing clear responsibilities, enabling local action, and active coordination which requires funding and identifying science's role.^55^^,^ 56 Indeed, the millennial generation, most familiar and dependent on Twitter and other social media platforms, also see themselves more as global citizens.^55^^,^ 56 As the next generation become disaster managers these electronic platforms, and their future iterations, will become commonplace worldwide as essential risk reduction tools in both developed and developing countries. The authors suggest that many more disaster response applications exist for the use of Twitter. For example, hashtags could be employed in search and rescue of individuals entrapped after an event such as an earthquake. If the individual or their family has access to a phone, they could use a (hashtag) #911 and turn GPS on to allow search and rescue teams to find the victims in real-time, further mitigating poor outcomes. The ‘power of Twitter’ has the potential to be a useful bimodal communication tool in real time that will benefit many at-risk populations.

## Limitations

A major limitation resides in concerns over Twitter spread of misinformation which could lead to tragic consequence before, during, and after a disaster. While this possibility exists with any form of communication, effective communicating and crowd sourcing has already demonstrated its contributions to legitimizing Twitter as a disaster response mechanism. Indiana University’s “truthy database”, created by researchers, is designed to detect misinformation and other social pollution that would corrupt vital information and its reliability as an effective disaster risk reduction communications tool.^38^ Further scrutiny is also a concern for the National Science Foundation’s creation of a web service that will monitor “suspicious memes”; however at present this effort is not focused on crises related activities, but on detecting “false and misleading ideas” with a primary focus on online political activity.^38^ The authors believe that effective competency based education and training curriculum and content has the best potential to assure the identification of a cadre of community and regional Twitter users to become effective and reliable partners in community-based communication. The use of local media user groups rather than GPS locations may introduce proxy population mismatch despite the sampling validation through GPS coordinates.

## Conclusions

Twitter is but one of many electronic communication tools available today and used by a worldwide population. This study describes the methodology by which Twitter was investigated as a possible communications tool for disasters and as an essential disaster risk reduction and management tool at the community level. By understanding how these various factors contribute to superspreading of messages, one can better optimize Twitter as a potential risk communication and disaster risk reduction tool. Parts II, III, and IV of this study further detail the technological and scientific base necessary for the community development of the tool for competency based Twitter assisted disaster management education and training.

## Competing Interests

The authors have declared that no competing interests exist.
